# Management of a Modern Operating Theatre

**Published:** 1903-05-02

**Authors:** A. Ferguson MacCallan


### Hospital Clinics and Medical Progress.

### MANAGEMENT OF A MODERN OPERATING THEATRE.

By A. Ferguson MacCallan, M.B., B.C. Camb., F.R.C.S. Eng.

(*Continued from page* 62.)

### The Management.

Having dealt with the disposition of the rooms and with some of the surgical impedimenta required to make the theatre a convenient and trustworthy place for the performance of surgical operations, I shall consider the outlines of the management of the theatre under the following headings :—

1. Preparation for the operation.
2. The operation.
3. Clearing up after the operation.

#### 1. *Preparation for the Operation.*

*a. Dressings, etc.*—The careful sterilisation of towels, swabs, and dressings is most important, and cannot be efficiently effected without proper apparatus. In large hospitals sterilisation is usually done in a large steam steriliser heated by a furnace and managed by an engineer who daily receives and purifies the dressings, etc., for the following day, which are sent down to him in a Schimmelbusch's kettle. The kettle is a nickel-plated box, about the size of a hat-box, which can be hermetically closed, but with an arrangement whereby it may be converted into a sieve-like box for the penetration of hot steam during the process of sterilisation.

In smaller hospitals where for the sake of economy the large steam steriliser is only used once a week, it is necessary to have a kettle for each day in the week. After sterilisation the boxes are closed and the contents remain sterile for an indefinite period if the boxes are placed in a clean room free from draughts and dust. Each kettle contains dressings, etc., for one day ; at the close of each day the box is refilled for resterilisation with any dressings which remain unused, together with fresh dressings to the necessary amount.

Theoretically it is best to remove dressings from the kettle as required for each operation ; practically it is often more convenient, in fact always in the case of the swabs, to transfer the contents of the kettle to carbolised glass jars. If the various articles for sterilisation are packed, each according to its kind, in lint bags with the mouth of the bag secured by a tape, each bag may be transferred *in toto* to a carbolised glass jar, by the aid of sterilised forceps, without any contamination. Towels, aprons, etc., may be left in the kettle until the end of the day, and withdrawn as needed.

In hospitals and nursing homes, where there is no installation of sterilisation plant, for a comparatively small outlay Schimmelbusch's dressings steriliser can be obtained, which can be easily worked by an intelligent nurse. Descriptions and estimates for this apparatus will be supplied by one of the larger firms of instrument makers. The general principle is the same in both varieties of steriliser; that is that noxious germs in the dressings are destroyed by the application of steam heated many degrees above the temperature at which steam is formed from boiling water.

*b. Ligatures and Sutures.*—These are usually at the present time composed of silk or silkworm gut. The latter is more easily prepared for use by boiling in water for 15 minutes and storing in absolute alcohol. Silk is more difficult to render sterile ; for this purpose the application of superheated steam in the dressings steriliser is necessary. During sterilisation the silk should be loosely wound on to a glass slide ; it should afterwards be rewound on to a glass bobbin, resterilised, and stored in absolute alcohol.

Another method of preparing silk for surgical purposes is by heating to 340° in olive oil ; the temperature is gauged by a thermometer, or in a more rough and ready fashion by dropping pellets of bread into the oil ; as soon as the pellet becomes brown the right temperature has been reached and should not be exceeded. The oil is removed after cooling by shaking in ether. The silk is then rewound, with all aseptic precautions, on to glass bobbins, and is stored in absolute alcohol.

The silk used for ligatures should, during the operation, be placed in the small wide-mouthed glass bottle in which it is kept on the glass table which supports the instrument-tray. Suitable lengths should be cut off it as required by the aid of sterilised scissors and forceps, which are used for nothing else, and are placed in a tray and covered with the same solution as is used for the instruments. The bobbin should never be touched except with the sterilised instruments.

The silkworm-gut sutures are kept in a round glass dish with a ground glass cover ; for removing them from this the same forceps may be used as are used for the ligatures. They are easily threaded into the needles which have previously been boiled.

*c. Instruments.*—All modern instruments can be sterilised by heat in the instrument steriliser. When proper precautions are taken neither the edges of the most delicate knives nor their ivory handles are destroyed. The varieties of apparatus designed are very numerous. The most convenient forms have electricity or gas as the heating agent. The fluid in the steriliser should be distilled water, and to this should be added pure carbonate of soda to the extent of 1 per cent. As the solution boils the water vaporises and must be replaced, but the soda need not be renewed. If the knives are placed in brass racks in the solution so as to prevent vibration during ebullition their edges will not be destroyed.

Previous to the operation the instruments are removed from the steriliser with a large pair of forceps to a porcelain tray. This tray is supposed to have been brought to as high a pitch of cleanliness as is possible by a nurse. The tray is flushed three or four times with boiling water, and is then filled with distilled water containing in solution $\frac{1}{2}$ per cent. of pure carbonate of soda and $\frac{1}{4}$ per cent. of carbolic acid. This solution has been boiled immediately before use. The object of the distilled water is to prevent the deposition of calcium salts on metal instruments : the sodium carbonate is to prevent the spotting of the instruments, which occurs when the salt is not used ; the carbolic acid is to retard the growth of organisms, which get access to the solution from the air.

*d. Lotions.*—These are most conveniently made and stored in large earthenware jars or pitchers fitted with tin caps. They must be heated gradually before the boiling water is put into them to compound the lotion. The glass barrels with gilt letters on them, which in many theatres delight the eye of the inexperienced observer, are inconvenient, expensive, and septic. The lotions are supplied to the surgeons in the particular kind of receptacle which is most convenient for each operation, whether it be porringer, bowl, or irrigator.

The water should be filtered through a porcelain filter before being used to compound the lotions in order to remove sand and other particles.

*e. Sponges.*—The old-fashioned Turkey sponges should be dispensed with. They are very expensive, and extremely difficult to sterilise. They should be replaced by pads of best absorbent wool wrapped and sewn up in fine gauze. When smaller sponges are required, small thin squares of wool may be used without any gauze covering. Any degree of moisture in the sponges may be obtained by damping them before sterilisation. If they are desired absolutely dry this may be easily effected where there is a large furnace-steriliser by drying after steam sterilisation, with hot air while still in the steriliser.

2. *The Operation.*

*a. The Hands.*—The hands of the operator, his assistant, and the nurses are much more difficult to approximate to a sterile condition than the other things which come into direct or indirect contact with the operation wound. It is probable that absolute sterility of the hands is impossible in spite of the numerous expedients, many of them painful, which have been devised in the attempt to attain that condition. The skin of some people is rendered rough, cracked, and eczematous by the use of strong antiseptics, and it is most important to avoid this condition, which is incompatible with sterility. The nails should be kept short and smooth, loose pieces of skin should be avoided about them, and there should always be a bottle of glycerine placed near the wash-basins. A convenient antiseptic application for the preservation of the skin is a mixture of equal parts of glycerine and water containing 5 per cent. of resorcin in solution. This should be applied while the hands are moist, well rubbed into the skin, and washed away by a stream of clean water before the hands are dried.

Nurses should be encouraged by the provision of good soap to wash their hands frequently, as the cheaper soaps are unpleasant to use and roughen the skin. There is an art in the washing of hands as in other things. The water should be pleasantly hot, the hands are immersed in it for a second or two, then a plentiful lather is made with the soap, and the hands should massage one another carefully; then the lather is washed away in the basin; the nail-brush, the bristles of which should not be too hard, after being soaped, should carefully be applied to the whole of the hand and especially to the nails; the lather is washed away in the basin and the nail-brush laid aside, a fresh lather massage is then done, and the lather is removed by a stream of tepid water from the tap. After this process has been thoroughly carried out, I do not consider the immersion of the hands in strong antiseptic solutions to be obligatory.

*b. Duties of Nurses in the Theatre.*—Each nurse in the theatre should have well-defined duties which should not be interchanged. If this is arranged carefully the fewer nurses there are in the theatre the better; three nurses would be sufficient for most operations, and two are often sufficient if they are capable and intelligent women. The duties of nurses are of course more numerous in hospitals where there are no student-assistants at the operation. In abdominal operations the counting of the sponges is the business of a nurse.

The method adopted for the handing of sponges to the chief assistant to the surgeon varies in different hospitals. Where there is no well-tried usage it will be found convenient for a nurse to remove from the glass jar, with a long pair of sterilised nickel-plated tongs each sponge as it is required, the top of the jar being replaced between each removal. In operations where rapidity is important, several sponges at a time may be placed ready to the chief assistant's hand on the sterilised towel which isolates the field of operation.

The used sponges should not be touched by the nurse with her hands, but should be thrown into a receptacle conveniently placed for them and at intervals emptied by a nurse into an enamelled iron pail which the theatre porter empties at intervals.

3. *Clearing up after the Operation.*

*a. The Floor.*—Blood and *débris* are removed by the theatre porter with a mop moistened in a bucket of 5 per cent. carbolic acid solution.

*b. The Instruments, etc.*—The instrument table is wheeled round to the steriliser. All delicate instruments are removed by the person who has charge of the instruments; the others in the tray are given to a nurse to clean in soap and water with a nail brush, previous to resterilisation, after which they are dried and polished with chamois leather. Delicate instruments are cleansed of blood and mucus under the cold tap, placed in the boiling water of the steriliser for a few seconds, removed and dipped into absolute alcohol, and then carefully dried with a very fine towel. A fine lawn handkerchief is the best thing for drying very delicate instruments.

If the operation has been of a definitely septic nature all instruments should be boiled for not less than 10 minutes, during the whole of which time the water should be in a state of full ebullition. After septic contamination it is very difficult to thoroughly purify trays and porringers which have been used during the operation. There is no certain method of effecting this except by boiling or sterilisation of superheated steam. In the case of very large instrument trays which have become septic and cannot easily be boiled, some authorities advise lavage for 15 minutes by boiling water from the tap and then filling the trays with 5 per cent. carbolic acid for many hours.

If the operation has been aseptic, the trays should be washed with soap and water and then placed in a stream of boiling water for a few minutes. They are then ready for use.

Porringers, etc., used for the reception of septic discharges or vomits should be marked plainly, and should be used for no aseptic purposes.

*c. Towels* after use should be thrown into an enamelled iron bucket, removed at the close of operations from the theatre to a place where they can remain until sent to the laundry. If the towels are septic they should be carbolised.

*d. Operations having finished*, the theatre must be cleaned in the usual way. During this process all movables should be removed to the theatre-nurses' room.

The above is a sketch from which may be elaborated the management of a first-class theatre. I have known theatres which appeared to the superficial observer to be everything that could be desired as regards asepsis, and yet when their management was investigated more closely, it was found in each case that owing to the carelessness of some person the whole costly apparatus was rendered of no effect.

It is under the careful management and constant care of some person in authority only, be it surgeon, medical superintendent, or matron, that a theatre can be kept up to the proper standard of asepsis. Of enormous importance also is the provision of a capable woman as theatre-nurse who willingly and intelligently strives to render her theatre as aseptic as it is possible to make it.

